# Relationship between teaching-research officers’ self-efficacy and occupational well-being: the mediating role of work engagement and the moderating role of organizational climate

**DOI:** 10.3389/fpsyg.2026.1737817

**Published:** 2026-08-03

**Authors:** Xiaoxiao Qu, Jianwen Guo, Lei Qiao, Mei Chen

**Affiliations:** 1 College of Education, Inner Mongolia Normal University, Hohhot, China; 2 College of Elementary Education, Capital University, Beijing, China; 3 Pu’er University, Pu’er, Yunnan, China

**Keywords:** occupational well-being, organizational climate, self-efficacy, teaching-research officer, work engagement

## Abstract

**Introduction:**

Despite previous studies indicating a strong correlation between self-efficacy and occupational well-being, the specific relationship and underlying processes remain unclear among teaching-research officers in primary and secondary schools within China’s ethnic regions.

**Method:**

This study established a moderated mediation model based on self-efficacy theory and resource conservation theory to investigate the relationship between self-efficacy and occupational well-being among teaching-research officers in China’s ethnic minority regions—the analysis aimed to clarify how work engagement mediates the association and assess the organizational climate’s moderating effect. A questionnaire survey was conducted by convenience sampling, including 240 teaching-research officers, and the mediation model with moderation was analyzed using SPSS and the Process macro plugin.

**Results:**

The findings indicated: (1) Self-efficacy positively predicted occupational well-being; (2) Work engagement mediated the relationship between self-efficacy and occupational well-being; (3) Organizational climate moderated the relationship between self-efficacy and occupational well-being. Specifically, compared to low organizational climate, high organizational climate significantly enhances the positive predictive effect of self-efficacy on occupational well-being; (4) Organizational climate moderated the relationship between work engagement and occupational well-being. Specifically, compared to high organizational climate, work engagement among teaching-research officers in low organizational climate demonstrated a much stronger positive predictive effect on occupational well-being.

**Conclusion:**

The findings reveal the underlying mechanisms between self-efficacy and occupational well-being. They also provide significant implications for educational administrators aiming to improve the occupational well-being of teaching-research officers in ethnic minority regions by optimizing their professional development and support systems.

## 1 Introduction

Teaching-research officers play a vital role in teacher education, and their professional well-being is closely tied to both their quality of life and their job performance (Viac and Fraser, 2020), and through fundamental responsibilities such as mentoring educators and developing curricula, they influence the effectiveness of frontline teachers and student academic achievement (Kraft and Hill, 2020; Kraft et al., 2018). However, this group encounters challenges from role ambiguity, reform pressures, and work complexity. Their levels of occupational well-being are not optimistic (Yin et al., 2020; Zhao, 2018). This issue is particularly pronounced in contexts where resources are scarce and collectivist culture prevails; for example, in the ethnic minority regions of southwest China, educators’ enthusiasm, emotional investment, and the quality of their work are significantly affected, and this can even lead to a desire to leave their jobs (Lee et al., 2024; Li J. et al., 2024), This situation not only affects their own physical and mental well-being but also raises concerns about the quality of teachers’ professional development and the academic achievement of students in underprivileged areas. Therefore, it is crucial to identify the factors that influence the professional well-being of instructional supervisors in different cultural contexts.

Self-efficacy theory, which posits that an individual’s belief in their ability to organize and execute specific tasks and achieve goals (Bandura, 1977), has been widely applied in research on educators’ well-being. For instance, multiple studies have confirmed that educators’ self-efficacy influences their professional well-being (Çiçek et al., 2025; Wang et al., 2024; Reppa et al., 2023; Zee and Koomen, 2016). The underlying mechanisms of the complex relationship between educational researchers’ self-efficacy and professional well-being require further investigation. Therefore, studying the mechanisms through which teaching-research officers’ self-efficacy influences professional well-being is of great significance and can help enhance individual well-being levels. How to improve the professional well-being of teaching-research officers’ has become a key focus in education and other fields.

The Conservation of Resources (COR) theory provides a theoretical framework for this. It posits that individuals actively seek to acquire, maintain, and protect valued resources (Hobfoll, 1989). As a crucial personal resource, self-efficacy can motivate individuals to more actively invest their resources—such as energy and time—into their work, thereby fostering a dynamic state characterized by vitality, dedication, and focused commitment (Schaufeli et al., 2002). The state of high engagement itself may enhance well-being by producing more successful experiences and social rewards (Caesens et al., 2014). Longitudinal studies demonstrate that educators’ self-efficacy predicts job satisfaction through work engagement (Granziera and Perera, 2019). So, does the mechanism by which self-efficacy influences well-being through work engagement still hold true in regions where resources are scarce and collectivist culture prevails? This study will test this hypothesis among a group of educational researchers in ethnic minority regions.

Furthermore, The boundaries of this mechanism are also unclear among educators in resource-scarce areas. The COR theory posits that the synergistic interaction between individual resources (such as self-efficacy) and environmental resources (such as organizational climate) collectively promotes occupational well-being; this is known as the resource-enhancing spiral effect (Hobfoll, 1989). As an environmental resource, organizational climate can both amplify and enhance the benefits of individual resources, and it can also suppress and deplete them (Hobfoll, 2011). A supportive organizational climate can offer essential environmental resources for individuals facing unique challenges, thus amplifying the beneficial effects of self-efficacy on well-being (Aldridge and Fraser, 2016). Conversely, a negative organizational climate may undermine this effect. Additionally, organizational climate may influence the relationship between work engagement and occupational well-being. In supportive climates, the benefits of work engagement may decrease slightly, as the environment contributes to well-being; In contrast, in unsupportive climates, work engagement may become the sole primary means for individuals to achieve well-being, thereby enhancing its predictive capacity while also increasing the risk of resource depletion (Xanthopoulou et al., 2009). However, it remains to be verified whether the organizational climate, as a boundary condition, still holds true in different cultural contexts, such as collectivist cultures.

Teaching-research officers working in China’s ethnic minority regions face a uniquely complex and challenging work environment, grappling with structural challenges such as relatively scarce educational resources and uneven educational development (Li and Huang, 2015). Based on this, this study primarily examines how the self-efficacy of teaching-research officers in China’s ethnic minority regions influences their occupational well-being through work engagement, as well as the moderating effects of organizational climate in this process. By constructing a moderated mediation model, the study aims to answer the following questions: First, does self-efficacy positively predict occupational well-being? Second, does work engagement play a mediating role? Third, does organizational climate moderate the relationship between self-efficacy and occupational well-being? By exploring the underlying mechanisms and boundary conditions of the relationship between self-efficacy and occupational well-being among teaching-research officers in minority regions, this study provides theoretical support and practical strategies for enhancing their job satisfaction, At the same time, it extends the testing of COR theory to resource-constrained educational contexts, verifying how different sociocultural contexts influence the interaction patterns between individuals and environmental resources. This provides scientific empirical evidence for educational administrators in underdeveloped regions to strengthen the development of the teacher and educator workforce through targeted interventions, thereby promoting regional educational equity and quality.

## 2 Literature review and hypotheses

### 2.1 Occupational well-being and self-efficacy

Occupational well-being encompasses the positive evaluations and feelings individuals derive from their work, and is divided into three dimensions: cognitive, motivational, and emotional (Xu et al., 2021; Coetzee et al., 2021). It incorporates the positive emotional state of career role satisfaction derived from workplace support and inclusiveness (Thompson and Bruk-Lee, 2021). It also includes cognitive evaluations of work, such as a sense of meaning and accomplishment, as well as optimal psychological functioning states in the workplace (Ryan and Deci, 2001). Existing studies suggest high levels of occupational well-being among educators improve work motivation and performance while promoting more positive emotional experiences (Gyeltshen and Beri, 2018). It effectively decreases teacher turnover rates (Parker et al., 2012).

Self-efficacy highlights the significance of personal beliefs when engaging in new or intricate tasks and managing challenges (Schwarzer, 2014). Self-efficacy serves as a crucial predictor of their occupational well-being (Zee and Koomen, 2016; Skinner et al., 2021; Bartosiewicz et al., 2022; Sun et al., 2022; Reppa et al., 2023; Lee et al., 2024; Li J. et al., 2024; Wang et al., 2025; Nas et al., 2026). Individuals exhibiting high self-efficacy are more inclined to recognize the significance of their work and enhance their experience of occupational well-being (Clegg and Spencer, 2007; Liu and Ren, 2010). When educators face challenges such as increased workload, pressure to reform, higher standards, and stricter accountability mechanisms (Fred et al., 2021; Cefai and Cavioni, 2013), their sense of well-being improves if they view their work as closely connected to their daily lives and as deeply meaningful (Brady and Wilson, 2021; O’Brien and Guiney, 2021). Consequently, educators necessitate a robust sense of efficacy to improve goal attainment and enhance overall well-being (Bermejo-Toro et al., 2016; Wang et al., 2025). Thereby, the subsequent hypotheses are presented:

*H1:* Teaching-research officers’ self-efficacy significantly and positively predicts their occupational well-being.

### 2.2 The mediating role of work engagement

Work engagement is characterized as a positive and fulfilling psychological state within the workplace. Its core components include vigor, which refers to possessing abundant energy and psychological resilience, alongside a willingness to exert effort and persevere; dedication, which encompasses feelings of passion, meaning, inspiration, and challenge related to work; and absorption, defined as being fully immersed in work, making it difficult to detach and perceive the passage of time (Schaufeli et al., 2002; Schaufeli and Bakker, 2004; Schaufeli, 2004). For educators who serve as mentors, organize teaching and research activities, and develop curricula, a strong commitment to their work is essential (Bakker and Demerouti, 2007; Bakker and Demerouti, 2008).

Self-efficacy is significantly associated with work engagement. Social cognitive theory suggests that self-efficacy and work engagement mutually reinforce each other (Granziera and Perera, 2019; Lent and Brown, 2006). Research also indicates that self-efficacy is a stable and significant precursor to work engagement (Xanthopoulou et al., 2007; Bresó et al., 2011; Uppathampracha and Liu, 2022; Haseli Songhori and Omrani Mehr, 2025). Individuals who perceive themselves as capable of completing work tasks tend to exhibit higher levels of energy, motivation, and willingness to contribute; in contrast, those who view themselves as incapable are more prone to disengage from efforts (Llorens et al., 2007; Lisbona et al., 2018). In addition, research also indicates that educators’ self-efficacy significantly enhances their work engagement in educational settings. Teachers exhibiting high self-efficacy are more adept at utilizing their strengths in response to diverse work stress sources, enhancing their work engagement (Klassen and Tze, 2014; Upadyaya et al., 2016; Granziera and Perera, 2019), and effectively reducing turnover intentions (Yerdelen et al., 2018). Longitudinal studies also demonstrate their evolving relationship. Initial self-efficacy is a significant predictor of subsequent work engagement (Simbula et al., 2011).

So, does an increasing level of work engagement further affect occupational well-being? A growing body of evidence indeed confirms that work engagement is closely linked to occupational well-being. Studies indicate that higher work engagement not only contributes positively to educators’ professional development (Lyons, 2006), but also directly improves their well-being (Rusu and Colomeischi, 2020; Fathi et al., 2024), thereby fostering more effective teaching behaviors (Soininen et al., 2023), and resulting in higher occupational well-being and a lower risk of burnout (Caesens et al., 2014; Bakker and Oerlemans, 2016; Hakanen et al., 2018; He et al., 2019).

Thereby, self-efficacy are essential antecedents that stimulate work engagement (Xanthopoulou et al., 2007). Work engagement is crucial for improving occupational well-being (Demerouti and Bakker, 2011). Some Researches have confirmed work engagement serves as a mediating factor between self-efficacy and occupational well-being (Gu, 2014; Rusu and Colomeischi, 2020; Zhou and Hou, 2025). However, the practical applicability of the teaching research officer’s work in regions characterized by scarce resources and a predominantly collectivist culture has yet to be verified. Therefore, this study proposes the following hypothesis:

*H2:* Teaching-research officers’ work engagement mediates the relationship between self-efficacy and occupational well-being.

### 2.3 The moderating influence of organizational climate

Organizational climate denotes members’ perceptions regarding the work environment (Rousseau, 1988). It reflects the collective comprehension of organizational policies, systems, practices, and procedures among members, as well as the rewarded behaviors (Schneider et al., 2013), encompassing four dimensions: management, work, learning, and interpersonal relationships (Pan and Sun, 2002). leadership climate, interpersonal climate, and work climate collectively form the work context that affects educators’ overall self-efficacy (Hoy and Woolfolk, 1993; Tschannen-Moran and Woolfolk Hoy, 2001; Weisel and Dror, 2006). Educators who perceive that schools offer sufficient resources and support exhibit increased self-efficacy (Skaalvik and Skaalvik, 2010; Malinen and Savolainen, 2016; Wilson et al., 2018) and enhance their confidence in teaching abilities (Zhang et al., 2023). Thus, organizational climate has a significant impact on educators’ self-efficacy (Tschannen-Moran and Hoy, 2007; Katsantonis, 2019).

Futhermore, Multiple studies demonstrate that a favorable organizational climate, characterized by supportive work environments and high-quality physical settings, is directly associated with educators’ occupational Well-being (Kusma et al., 2012; Rombaoa Tanaka et al., 2020; Kwon et al., 2021). It notably improves theirs’ well-being (Thapa et al., 2013; Yusoff and Tengku-Ariffin, 2020), decreases their turnover intention (Ryu et al., 2020). Thereby, studies indicate demonstrates that a well-structured, supportive, and collaborative environment improves educators’ resilience in overcoming challenges and enhancing the positive impact of personal resources on outcomes like innovative behaviors (Gray et al., 2017; Lin et al., 2021; Loh et al., 2019; Han et al., 2023) and decrease burnout rates (Grayson and Alvarez, 2008; Idris and Dollard, 2014). Consequently, the subsequent hypothesis may be proposed:

*H3:* Organizational climate moderates the relationship between self-efficacy and occupational well-being among teaching-research officers. Specifically, under high organizational climate conditions, self-efficacy exerts a more substantial positive predictive effect on job satisfaction. In contrast, under low organizational climate conditions, the positive predictive effect of self-efficacy on job satisfaction is weakened.

The organizational climate may moderate the relationship between work engagement and occupational well-being. According to the Conservation of Resources theory (COR), while in a positive resource state, work engagement does not inherently improve occupational well-being; somewhat, its impact is moderated by environmental resources (Hobfoll, 2011). The returns individuals receive from resource investments primarily depend upon environmental support. Supportive environments promote a “resource gain spiral.” Conversely, resource-depleted environments can lead individuals to may experience a “resource depletion spiral” (Hobfoll et al., 2018). Existing studies indicate that when environmental resources, including social support, autonomy, and team atmosphere, are plentiful, invidiuals’ work engagement correlates with enhanced daily task performance (Xanthopoulou et al., 2009; Beal et al., 2005). Conversely, some studies have shown that when environmental resources are scarce, individuals may deplete their personal resources without being able to replenish them, thereby reinforcing the link between resource depletion and negative outcomes (Halbesleben et al., 2009). Thereby, organizational climate has been verified to significantly influence the work engagement of teaching-research officers (Yang and Hu, 2021). Therefore, in a favorable organizational climate, the work engagement of teaching-research officers receives full organizational resource support. But, in this context, the environment significantly contributes to sources of well-being, which may reduce the marginal benefits of work engagement and weaken its predictive capacity for occupational well-being. In low organizational climates, teaching-research officers are required to invest extra personal resources to address environmental resource limitations. Work engagement serves as a crucial pathway for individuals to achieve well-being, thereby enhancing its predictive significance. This approach, however, poses a risk of resource depletion, as it compensates for environmental resource deficiencies through “self-sacrifice” and “dedication” (Schaufeli, 2016). Therefore, the subsequent hypothesis is proposed:

*H4:* Organizational climate moderates the relationship between work engagement and occupational well-being among teaching-research officers. Specifically, work engagement exerts a more substantial positive predictive effect on occupational well-being under low organizational climate conditions. In contrast, under high organizational climate conditions, the positive predictive effect of work engagement on occupational well-being is attenuated.

**Figure 1:**
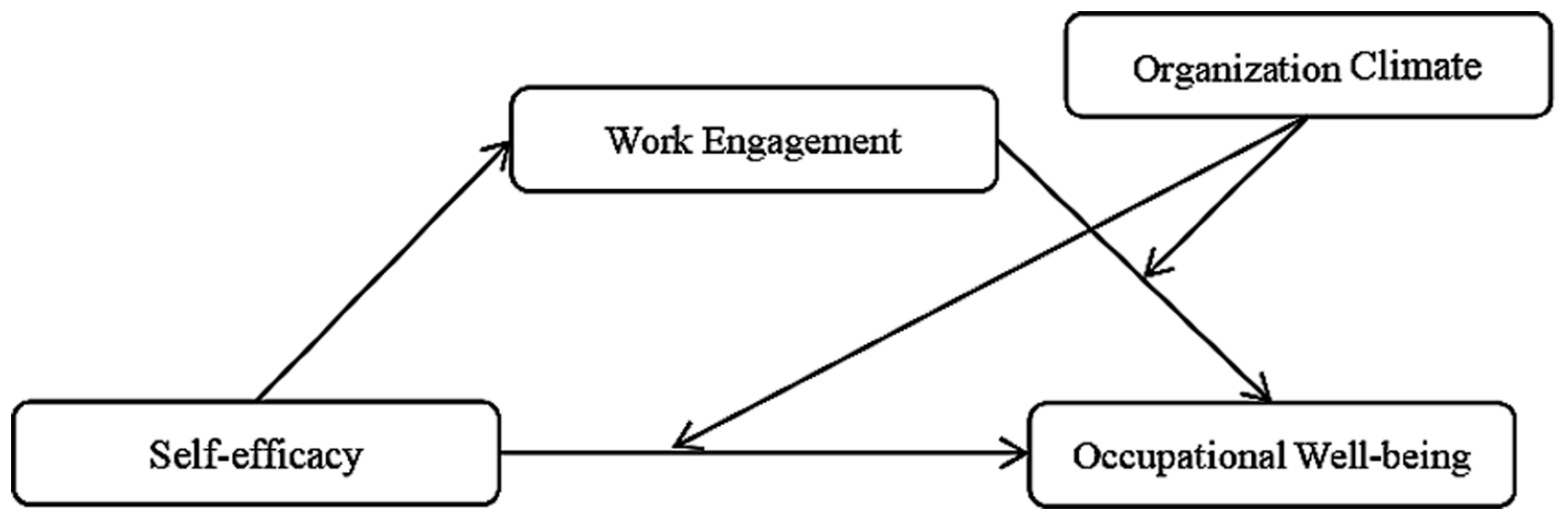
Hypothesis model of self-efficacy, work engagement, organizational climate, and occupational well-being among teaching–research officers.

In summary, we constructed the mediation model for this study, as shown in Figure 1.

## 3 Methods

### 3.1 Participants

This study focuses on teaching-research officers in the ethnic regions of Southwest China. Given this group, situated within the government education administration system, is marked by high professionalization, limited size, and low accessibility, which complicates the acquisition of a large-scale random sample. Therefore, this study utilized a combination of purposive and snowball sampling methods to recruit participants from specialized teaching-research officers integrated within the administrative framework of ethnic regions in Southwest China. Between April 25 and June 10, 2024, access to municipal-level teaching research institutions was obtained via official channels of the Guizhou Provincial Department of Education. After explaining the research objectives, we obtained their consent. Initially, the questionnaire was distributed online to municipal-level teaching-research institutions in Guizhou, which serve ethnic regions. Simultaneously, we encouraged teaching-research officers who received the questionnaire to disseminate it with eligible peers, thereby generating a snowball effect to enhance sample coverage. All procedures in this study were approved by the Institutional Review Board (IRB) of the authors’ affiliated institution.

A total of 254 questionnaires were collected for the study. Excluding questionnaires with only extreme responses and those completed in under 2 min resulted in 229 valid questionnaires, achieving a response rate of 90.16%. The survey maintained anonymity, and participants were informed of the research objectives and their right to participate voluntarily. A post-hoc power analysis was conducted using G*Power software, referencing Cohen’s (2013) standards to evaluate the adequacy of the sample size in terms of statistical power. The analysis indicated an effect size of f^2^ = 0.15 (medium), *α* = 0.05, and 8 predictor variables (demographic, main, and interaction terms), based on a sample size of *N* = 229. The findings demonstrated that the statistical power of the study (1-*β*) was 0.99, which notably surpasses the conventional standard of 0.80. This suggests that the existing sample size is capable of identifying moderate or larger effects within the model, with statistical evidence affirming its adequacy. All procedures received approval from the Institutional Review Board (IRB) at the authors’ university. The sample comprised 89 males (38.87%) and 140 females (61.14%); The age distribution is as follows: 3 participants (1.31%) are aged 20–25, 85 participants (37.12%) aged 26–35, 79 participants (34.50%) aged 36–45, 50 participants (21.83%) aged 46–55, and 12 participants (5.24%) aged 55 and above. Regarding teaching experience: 25 individuals (10.92%) had 5 years or less; 54 individuals (23.58%) had 6–10 years; 39 individuals (17.03%) had 11–15 years; 26 individuals (11.35%) had 16–20 years; 39 individuals (17.03%) had 21–35 years; and 46 individuals (20.09%) with 26 or more years of experience. In terms of professional titles, 15 individuals (6.55%) were unclassified, 46 individuals (20.09%) held junior titles, 99 individuals (43.23%) held intermediate titles, and 69 individuals (30.13%) held senior titles. Regarding educational qualifications, 31 individuals (13.54%) possessed associate degrees or lower, 194 individuals (84.71%) held bachelor’s degrees, and four individuals (1.75%) attained master’s degrees or higher.

### 3.2 Measures

All quantitative measures used in this study were derived from established English or Chinese scales. To ensure their applicability, surface validity, and content validity within the specific cultural context and occupational group (educational-research officers in ethnic regions) of this study, we followed a standardized cross-cultural adaptation process: First, two bilingual psychology researchers proficient in both Chinese and English conducted independent translations and back-translations, reconciling discrepancies through consultation. Second, three experts in educational psychology and two experienced teaching researchers reviewed the clarity, content relevance, and cultural appropriateness of the initial Chinese-language items. Based on their feedback, we refined the wording of certain items to better align with the work context of teaching researchers, for example, specifying “workplace” as “teaching and research environment”.

#### 3.2.1 General self-efficacy scale

The self-efficacy of teaching-research officers was evaluated using the General Self-Efficacy Scale, created by Schwarzer et al. (1997) and validated by Wang et al. (2001). The scale consists of 10 items evaluated on a 5-point Likert scale ranging from 1 (strongly disagree) to 5 (strongly agree), with elevated scores signifying increased self-efficacy. Representative items include: “If I put in the necessary effort, I will be capable of resolving the majority of the challenging issues in my teaching and research endeavors.” The overall Cronbach’s alpha coefficient was 0.911. Confirmatory factor analysis produced positive outcomes: X^2^/df = 2.122, CFI = 0.979, IFI = 0.980, RMSEA = 0.070, SRMR = 0.032.

#### 3.2.2 Work engagement scale

Work engagement was assessed utilizing the scale developed by Schaufeli et al. (2002) and subsequently revised by Zhang and Gan (2005), known as the Utrecht Work Engagement Scale (UWES). It also demonstrated reliability and validity in the studies conducted by Chinese teaching-research officers (Yang and Hu, 2021). The scale consists of 17 items distributed among three dimensions: vigor, dedication, and focus, with Cronbach’s *α* coefficients of 0.907, 0.890, and 0.903, respectively. All items are evaluated using a 5-point Likert scale ranging from 1 (strongly disagree) to 5 (strongly agree), with higher scores reflecting increased work engagement. A representative item is: “While engaging in teaching and research responsibilities, I feel a strong sense of motivation.” The overall Cronbach’s alpha coefficient was 0.954. Confirmatory factor analysis yielded positive outcomes: X^2^/df = 2.194, CFI = 0.960, IFI = 0.960, RMSEA = 0.072, SRMR = 0.040.

#### 3.2.3 Organizational climate scale

The organizational climate scale was developed by Chinese scholars Pan and Sun (2002) and subsequently revised by Zhao (2022). This scale has demonstrated strong reliability and validity in previous studies. The final scale comprises 14 items distributed across four dimensions: management atmosphere, teaching atmosphere, learning atmosphere, and interpersonal atmosphere. The Cronbach’s α coefficients were 0.897, 0.847, 0.807, and 0.906, respectively. The scoring used a 5-point Likert scale ranging from 1 (strongly disagree) to 5 (strongly agree), with higher scores indicating better organizational atmosphere. A representative item is: “All rules and regulations are well-established, enabling adherence to established procedures.” The overall Cronbach’s alpha coefficient was 0.947. Confirmatory factor analysis yielded favorable results: X^2^/df = 2.316, CFI = 0.964, IFI = 0.964, RMSEA = 0.076, SRMR = 0.038.

#### 3.2.4 Occupational well-being scale

The Occupational Well-being Scale was developed by Chinese researchers Zhao et al. (2012) and Zhao (2012). This scale consists of 22 items rated on a 5-point Likert scale ranging from 1 (strongly disagree) to 5 (strongly agree), with higher scores indicating greater occupational well-being. Representative items include “My profession is respected by society.” The overall Cronbach’s alpha coefficient for this scale is 0.939. Confirmatory factor analysis yielded satisfactory results: X^2^/df = 1.666, CFI = 0.971, IFI = 0.971, RMSEA = 0.054, SRMR = 0.049.

#### 3.2.5 Demographic information

The demographic information included gender, years of teaching experience, age, professional title, and other relevant factors. Previous studies indicate a significant correlation between teachers’ occupational well-being and factors such as gender, years of teaching experience, age, professional title, and educational background (Barbieri et al., 2019; Zuo and Wang, 2023). Therefore, this study incorporates these variables into basic statistical information and accounts for them in subsequent analyses to prevent their influence on research outcomes.

### 3.3 Data analysis

This study initially employed SPSS 27.0 to conduct common method bias, descriptive statistics, and Pearson correlation analyses on the data. Subsequently, to reduce multicollinearity between predictors and their interaction terms all data were standardized and analyzed by Hayes (2013) to evaluate hypotheses. Model 4 investigated the mediating role of work engagement in the relationship between self-efficacy and occupational well-being, and the bias correction percentile Bootstrap method with 5,000 resamples was used to test the significance of the mediation effect. Model 15 assessed the moderating effect of organizational climate. The Bootstrap method was also ultimately utilized for testing. If the 95% confidence interval included 0, the result was considered statistically insignificant.

## 4 Results

### 4.1 Common method bias test

The standard method bias was analyzed due to the data’s reliance on subjective reports from teaching-research officers. First, we conducted a preliminary analysis employing Harman’s single-factor method. The results showed that nine factors possessed eigenvalues exceeding one before rotation. The initial factor accounted for 38.785% of the variance, falling short of the 40% critical threshold (Podsakoff et al., 2003; Tang and Wen, 2020), indicating that the concern of common method bias is not significant. Secondly, to further strengthen validation, we integrated the four factors from the study into a single-factor model and evaluated it using Confirmatory Factor Analysis (CFA). The findings indicated that the single-factor model exhibited an inadequate fit (X^2^/df = 4.121, CFI = 0.508, IFI = 0.511, RMSEA = 0.117, SRMR = 0.123), further suggesting the absence of significant method bias. Additionally, we performed multicollinearity diagnostics as an adjunct. The results showed that the variance inflation factors (VIF) for the predictor variables were all below 5 (2.228, 2.267, 1.242), and the tolerances were all above 0.1 (0.449, 0.441, 0.805), thereby eliminating the presence of severe multicollinearity concerns.

### 4.2 Descriptive statistics and correlation analysis

Table 1 presents the means, standard deviations, and correlation analysis of relevant variables. The Pearson correlation analysis reveals: Demographic variables, including age, years of teaching experience, and professional title, exhibited significant correlations with self-efficacy (r = 0.163*, 0.160*, 0.139*); Additionally, age, years of teaching experience, and professional title were significantly correlated with work engagement (r = 0.198**, 0.190**, 0.173**). There were significant positive correlations at the *p* < 0.01 and *p* < 0.05 level among teaching-research officers’ self-efficacy, work engagement, organizational climate, and occupational well-being, with correlation coefficients ranging from 0.402 to 0.778. Self-efficacy among teaching-research officers showed significant positive correlations with work engagement, school organizational climate, and occupational well-being (r = 0.735, *p* < 0.01; r = 0.402, *p* < 0.01; r = 0.509, *p* < 0.01). Work engagement demonstrated significant positive correlations with organizational climate and occupational well-being (r = 0.419, *p* < 0.01; r = 0.588, *p* < 0.01). Additionally, organizational climate revealed a significant positive correlation with occupational well-being (r = 0.778, *p* < 0.01).

**Table 1:** Means, standard deviations, and correlations of the main variables (*N* = 219).

Variables	*M ± SD*	1	2	3	4	5	6	7	8	9
1. Gender	1.611 ± 0.48	1								
2. Age	2.926 ± 0.92	—	1							
3. Teaching age	3.603 ± 1.71	−0.128	0.894^*^	1						
4. Title	2.969 ± 0.87	−0.028	0.660^*^	0.733^**^	1					
5. Educational background	1.882 ± 0.37	0.156^*^	—	—	0.043	1				
6. Self-efficacy	3.173 ± 0.68	−0.119	0.163^*^	0.160^*^	0.139^*^	−0.011	1			
7. Work engagement	3.456 ± 0.71	−0.042	0.198^*^	0.190^**^	0.173^*^	0.047	0.735^*^	1		
8. Organizational Climate	3.81 ± 0.61	−0.019	0.024	0.022	0.03	−0.041	0.402^*^	0.419^*^	1	
9. Occupational Well-being	3.711 ± 0.56	−0.03	0.034	0.058	0.022	−0.021	0.509^*^	0.588^*^	0.778^*^	1

M = mean, SD = standard deviation. Gender: 1 = male, 2 = femal; age: 1 = 20–25 years old, 2 = 20–35 years old, 3 = 36–45 years old, 4 = 46–55 years old, 5 = over 55 years old; Teaching age: 1 = less than 5 years, 2 = 6–10 years, 3 = 11–15 years, 4 = 16–20 years, 5 = 21–25 years, 6 = over 26 years; title:1 = unrated, 2 = junior title, 3 = intermediate title, 4 = senior title; Educational background:1 = college diploma or below, 2 = bachelor’s degree, 3 = master degree. ^***^p < 0.001, ^**^p < 0.01, ^*^p < 0.05.

### 4.3 Mediation analysis

Model 4 analyzed the mediating effect of work engagement on the relationship between self-efficacy and occupational well-being, while controlling for demographic variables including gender and years of teaching experience. The results are presented in Table 2. First, self-efficacy was a significant predictor of occupational well-being (*β* = 0.540, t = 9.110, *p* < 0.001). Hypothesis 1 was validated. Second, self-efficacy was a significant predictor of work engagement (*β* = 0.726, t = 15.659, *p* < 0.001). Third, with work engagement as a mediating variable, self-efficacy significantly impacted job satisfaction (β = 0.165, t = 2.083, *p* < 0.05), and work engagement itself significantly predicted job satisfaction (β = 0.516, t = 0.079, *p* < 0.001). The bias-corrected bootstrap results demonstrated that the direct effect of self-efficacy on job satisfaction [0.003, 0.317], the mediating effect of work engagement [0.268, 0.483], and the total effect [0.517, 0.742] were excluded from the Bootstrap 95% confidence interval, indicating that work engagement mediates the relationship between self-efficacy and occupational well-being. Hypothesis 2 is supported, with the mediating effect accounting for 69.44% of the variance.

**Table 2:** Mediation effect of work engagement.

Outcome variable	Occupational well-being (module 1)			Work engagement (module 1)			Occupational well-being (module 3)		
Predictor variable	β	se	t	β	se	t	β	se	t
Self-efficacy	0.540	0.059	9.110^***^	0.726	0.046	15.659^***^	0.165	0.079	2.083^*^
Work engagement							0.516	0.079	6.524^***^
*R^2^*	0.286			0.563			0.403		
*F*	9.759^***^			31.288^***^			14.706^***^		

Bias-corrected bootstrap test of the intermediary effect					
	Effect	BootSE	BootLLCI	BootULCI	Percentage
Indirect effect	0.375	0.055	0.268	0.483	69.44%
Direct effect	0.165	0.079	0.009	0.320	30.56%
Total effect	0.540	0.058	0.517	0.742	100%

^***^p < 0.001, ^*^p < 0.01, *p* < 0.05.

### 4.4 Moderated mediation analysis

**Figure 2:**
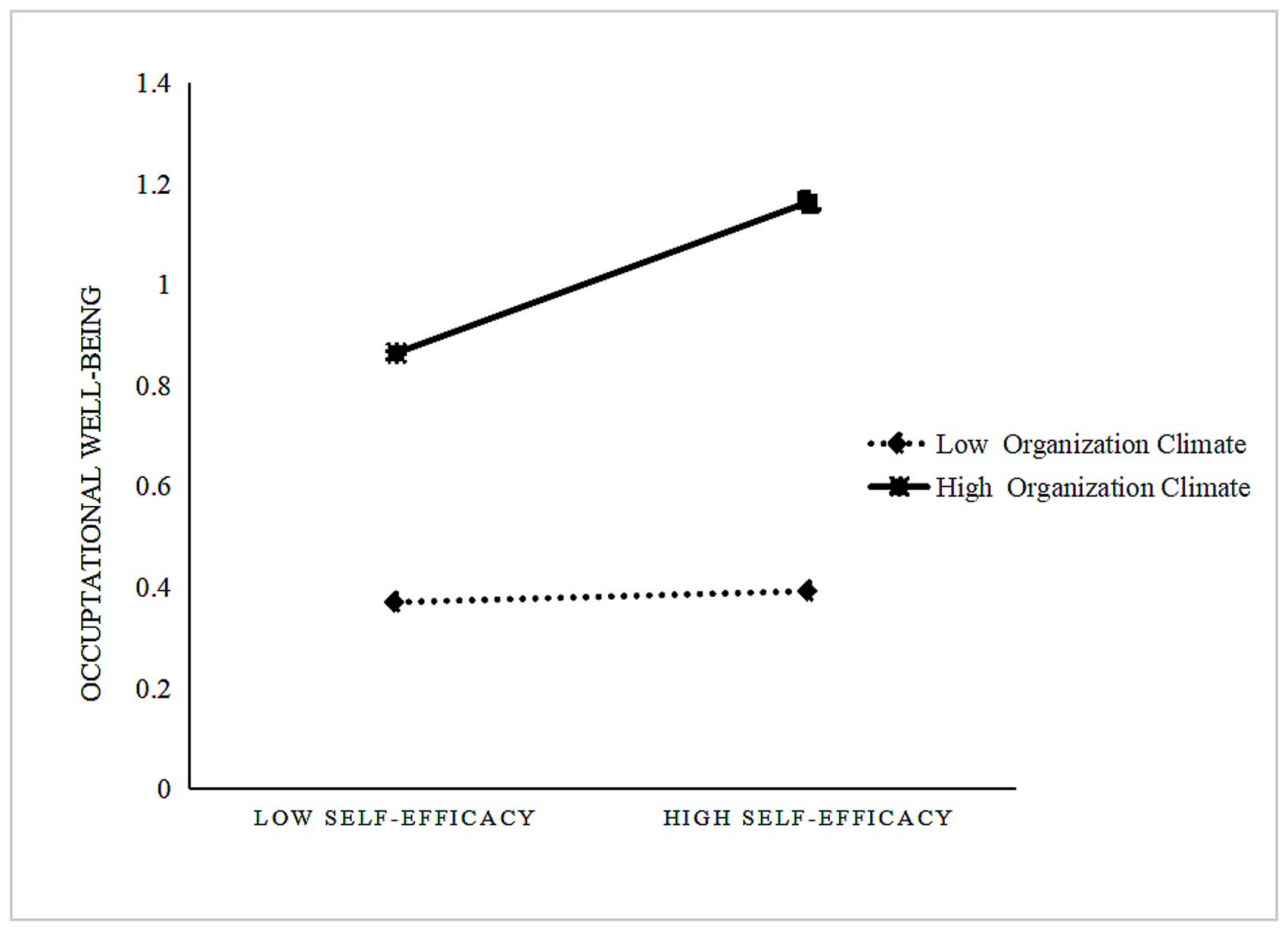
Organization climate as a moderator between self-efficacy and occupational well-being.

**Figure 3:**
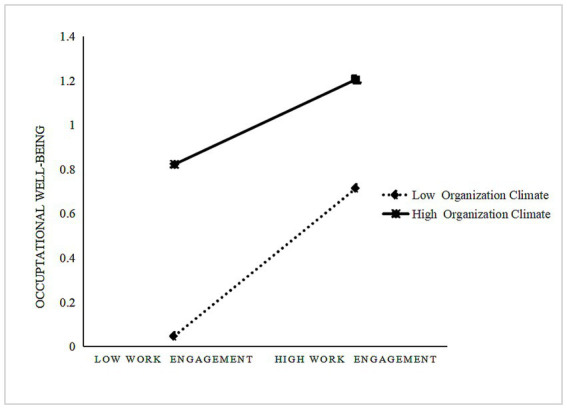
Organization climate as a moderator between work engagement and occupational well-being.

Model 14 tested the moderating effect of organizational climate, while accounting for demographic variables including gender and years of teaching experience (Table 3). The interaction between self-efficacy and organizational climate significantly and positively predicted occupational well-being (β = 0.139, t = 2.755, *p* < 0.01), thereby confirming Hypothesis 3–1. The interaction between work engagement and organizational climate negatively predicted occupational well-being (β = −0.142, t = −2.363, *p* < 0.05), confirming Hypothesis 3–2. A detailed analysis of the moderating effects of organizational climate on the relationship between self-efficacy and occupational well-being, as well as work engagement and occupational well-being, revealed the following through simple slope analysis. Figure 2 illustrates that in the high-scoring group of organizational climate (M + 1SD), self-efficacy significantly and positively predicted occupational well-being (β = 0.149, t = 2.133, *p* < 0.05), with a confidence interval of [0.011, 0.287]. Nevertheless, at a low organizational climate (M-1SD), the influence of self-efficacy on occupational well-being discontinued to be significant (β = −0.128, t = −1.571, *p* > 0.05), with a confidence interval of [−0.289, 0.033]. This indicates that a favorable organizational climate enhances the positive influence of self-efficacy on occupational well-being. Figure 3 illustrates that in the low organizational climate group (M-1SD), work engagement significantly and positively predicted occupational well-being (β = 0.476, t = 5.236, *p* < 0.001), with a confidence interval of [0.297, 0.655]. However, in instances of elevated organizational climate (M + 1SD), work engagement remained a significant positive predictor of occupational well-being (β = 0.191, t = 2.513, *p* < 0.05), with a confidence interval of [0.014, 0.341]. Nevertheless, this predictive effect was significantly less notable than in the low organizational climate condition.

**Table 3:** Moderating effect of organizational climate.

Outcome variable	Work engagement (module 1)			Occupational well-being (module 2)		
Predictor variable	β	se	t	β	se	t
Self-efficacy	0.726	0.046	15.659^***^	0.011	0.057	0.185
Work engagement				0.334	0.058	5.723^***^
Organization climate				0.633	0.043	14.69^***^
Self-efficacy × Organization climate				0.139	0.050	2.755^**^
Work engagement × Organization climate				−0.142	0.060	−2.363^*^
*R2*	0.563			0.709		
*F*	31.287^***^			40.200^***^		

Conditional effect							
Moderating variable	Level of moderating variables	β	se	t	*p*	95%CI	
Self-efficacy × Organization climate	M-1SD	−0.128	0.082	−1.571	0.118	−0.289	0.033
	M	0.011	0.057	0.185	0.853	−0.102	0.123
	M + 1SD	0.149	0.07	2.133	0.034	0.011	0.287
Work engagement × Organization climate	M-1SD	0.476	0.091	5.236	0	0.297	0.655
	M	0.334	0.058	5.723	0	0.219	0.449
	M + 1SD	0.191	0.076	2.513	0.013	0.041	0.341

^***^p < 0.001, ^**^p < 0.01, ^*^p < 0.05.

## 5 Discussion

This study focuses on the Chinese context to investigate the underlying mechanisms and boundary conditions by which self-efficacy affects occupational well-being among teaching-research officers in ethnic regions, within increasing scholarly attention to the professional lives of teacher educators (Kelchtermans et al., 2018). The study constructs and tests a moderated mediation model, providing substantial theoretical and empirical support for the factors influencing occupational well-being among teaching-research officers in China’s ethnic regions, while also presenting new evidence for applying and expanding self-efficacy theory and resource conservation theory in educational administration. Findings indicate that teachers’ self-efficacy directly and positively predicts occupational well-being and has indirect effects through the partial mediation of work engagement. Furthermore, organizational climate moderates the relationship between self-efficacy and occupational well-being and between work engagement and occupational well-being. These insights provide essential direction for systematically developing a framework to enhance occupational well-being for teachers in China’s ethnic regions.

### 5.1 Direct effect of self-efficacy on occupational well-being

The findings support Hypothesis 1, indicating that self-efficacy among teaching-research officers is a significant positive predictor of their occupational well-being. This discovery closely corresponds with findings from studies on teacher populations (Klassen et al., 2011a; Klassen et al., 2011b; Sun et al., 2022; Reppa et al., 2023; Billett et al., 2023; Lee et al., 2024). For teaching-research officers in China’s resource-constrained ethnic regions, self-efficacy appears to be a key psychological resource associated with navigating complex professional challenges. Individuals with high self-efficacy are more likely to appraise challenges as manageable and to maintain confidence in in their ability to overcome obstacles. The pattern is associated with a greater sense of accomplishment and higher levels of overall well-being. Conversely, those with low self-efficacy tend to report feelings of helplessness and a lack of confidence, which are linked to lower well-being (Bandura, 1997). This study found that teaching-research officers confident in their instructional research and mentoring abilities tended to demonstrate superior emotional and stress management skills. These competencies were associated with sustained positive work engagement and, ultimately, higher occupational well-being. This finding underscores that for teaching-research officers, robust self-efficacy in key professional domains is associated with more adaptive psychological functioning and positive work outcomes (Yang and Hu, 2021). This finding strongly supports the positive psychology perspectives, suggesting that self-efficacy as a positive psychological resource that significantly predicts the occupational status of teaching-research officers in ethnic regions.

Based on these associations, we propose systematically incorporating the cultivation of self-efficacy into the professional development of this group to enhance their occupational well-being by strengthening teaching-research officers’ self-efficacy. Self-efficacy primarily derives from mastery experiences (individual experiences of success or failure), vicarious experiences, verbal persuasion, and physiological arousal (Bandura, 1993). We propose four specific cultivation pathways: Initially, teaching research institutions refine task design to enhance opportunities for teaching-research officers to succeed and serve as role models. Secondly, establishing professional learning communities for teaching-research officers to observe exemplary peers and model cases may enhance of familiarity with the vicarious experiences of their colleagues. Third, positive feedback mechanisms from leaders, peers, and frontline teachers could be established to ensure ongoing psychological support through authentic assessment and acknowledgment. Fourth, teaching-research officers could be assisted in maintaining a positive mindset and calmly addressing challenges through stress management training and incentives. Participation in CPD programs has been linked to improved teaching-research officers’ efficacy and work engagement (Yin et al., 2020).

### 5.2 The mediating role of work engagement

Our study confirms that work engagement partially mediates the relationship between self-efficacy and occupational well-being, affirming Hypothesis 2. This finding comprehensively reveals the operational mechanism of work engagement among teaching-research officers in ethnic regions for the first time. It provides empirical support for COR theory’s “resource-enhancing spiral” principle. Specifically, as a personal resource, self-efficacy closely links to work engagement, which in turn is associated with greater occupational well-being. The mediation model in this study reveals a dual pathway:

First, self-efficacy significantly and positively predicted work engagement (path a). This is consistent with previous research (Yang and Hu, 2021). The resource investment principle in COR theory can effectively predict employee behavior (Halbesleben et al., 2009). Individuals actively strive to acquire, sustain, and preserve self-efficacy as a vital psychological resource. Educational researchers with elevated self-efficacy exhibit strong confidence in their teaching, research, and instructional guidance capabilities. Consequently, they tend to perceive their energy and cognitive resources as renewable assets instead of limited commodities. The cognitive framework is associated with proactive resource investment behaviors, in which individuals commit themselves wholeheartedly to complex work challenges to achieve greater resource returns, which is consistent with elevated levels of work engagement (Hobfoll, 1989).

Second, work engagement significantly and positively predicted occupational well-being (path b). This is consistent with prior research (Bakker and Oerlemans, 2016; Hakanen et al., 2018). Educators engaged in their profession have supplementary resources that positively impact physical and mental well-being (Airila et al., 2014; Seppälä et al., 2012). Work engagement is a favorable condition characterized by vitality, voluntary dedication, and complete commitment (Schaufeli et al., 2002). For educational researchers in ethnic regions, substantial resource investment produces greater returns, including superior research outcomes, peer recognition, and professional success, which are linked to enhance occupational well-being (Sinclair et al., 2024).

Finally, The study supports the hypothesis that work engagement significantly mediates between the self-efficacy of teaching-research officers and occupational well-being. It broadens the applicability of the Conservation of Resources theory (COR), which posits resource depletion rather than acquistion as its fundamental principle, arguing that any behavior associated with resource exhaustion is linked to negative psychological states in induviduals (Hobfoll et al., 2018). However, this study offers a complementary perspective on COR theory, suggesting that proactive resource investment behaviors are associated with positive resource gains, induce positive psychological states in individuals, and improve their well-being (Hobfoll, 1989). Therefore, work engagement serves as the key variable that transforms the teaching-research officers’ self-efficacy into occupational well-being. This conclusion suggests that, while training in self-efficacy is essential, equal attention must be devoted to maintaining and supporting teaching-research officers’ work engagement.

### 5.3 The moderating influence of organizational climate

The findings confirm the moderating influence of organizational climate, revealing its essential boundary condition. First, Hypothesis 3 is validated: organizational climate moderates the correlation between self-efficacy and occupational well-being, consistent with previous research (Wang et al., 2025). The Conservation of Resources Theory (COR), posits that resources are not isolated entities but are situated within an ecological context resembling a caravan route. This context can support resources while posing constraints that may impede their development and preservation (Hobfoll et al., 2018). The organizational climate functions precisely as a resource caravan route. In a favorable organizational climate, the self-efficacy of teaching-research officers in ethnic regions significantly and positively predicts their occupational well-being. The principal reason is that in high organizational climate conditions, individual resources synergize with environmental resources to obtain new resources, collectively fostering the development of a resource gain spiral. Research indicates that when administrative departments establish conducive working environments and conditions for educators, teachers may be inclined to extend their working hours in such settings (Waters, 2013). In contrast, within environments characterized by a low organizational climate, the significance of self-efficacy diminishes. The principal reason is that the personal resources such as confidence and effort among teaching-research officers are suppressed or even completely negated. As a result, these resources fail to produce the anticipated returns. For teaching-research officers in ethnic minority regions, they frequently confront complex circumstances such as disparate educational advancement. The failure to invest resources constitutes a loss of resources. Ultimately, it may be associated with a cycle of resource depletion (Hobfoll et al., 2018). Studies indicate that individuals in supportive roles encounter heightened psychological distress when they require support themselves (Hobfoll and London, 1986; Riley and Eckenrode, 1986). Furthermore, research confirms that unnecessary routine, managerial, and administrative responsibilities are more likely to foster negative attitudes, thereby undermining their occupational well-being (Mohan and Ashok, 2011). For instance, teachers at a Malaysian public school expressed dissatisfaction with their school due to inadequate physical conditions and work environment, which affected their work engagement (Knox, 2011). For teaching-research officers who fulfill both professional and administrative functions (Song, 2012), administrative responsibilities substantially diminish the resources that are designated for professional guidance. Consequently, despite possessing high self-efficacy, teaching-research officers find it challenging to attain a sense of value and fulfillment in their work.

Second, Hypothesis 4 is also confirmed. The organizational climate significantly moderates the correlation between work engagement and occupational well-being among teaching-research officers. Specifically, in environments with lower organizational climate, the positive predictive effect of work engagement on occupational well-being among teaching-research officers is pronounced than in environments with higher organizational climate. A plausible explanation is that teaching-research officers have access to various collective resources in high organizational climates, including organizational support, peer trust, and leadership recognition. Their sources of well-being are diverse, enabling them to attain satisfaction without necessitating exceptionally high levels of individual work engagement. As a result, the marginal benefits of work engagement decrease, undermining its positive predictive effect. In contrast, in low organizational climates characterized by limited collective resources, teaching-research officers are compelled to deplete personal resources (increase work engagement) to “compensate” for the deficiency of organizational resources necessary for sustaining work effectiveness, thereby maintaining basic professional well-being. This sense of well-being is particularly likely to manifest in the pursuit of a sense of accomplishment and meaning in work, rather than merely maintaining job satisfaction. This compensation establishes a more direct relationship between work engagement and occupational well-being. Work engagement is the primary pathway to achieving occupational well-being. This further validates the conclusion that the predictive effect is more significant in such climates. Nonetheless, this compensation effect represents a forced resource-depletion strategy. In the long term, it may result in exhaustion, threats to self-esteem, and subsequently, adverse coping mechanisms (SchiSnpflug, 1985), potentially culminating in burnout or workaholism (Bakker et al., 2011), ultimately diverging from initial organizational goals (Yilmaz et al., 2014). Furthermore, this conclusion is related to China’s collectivist cultural context. China has long been profoundly shaped by Confucian traditions and cultural thought, particularly evident in its strong collectivist orientation-a spirit advocating wholehearted dedication and selfless devotion to collective interests (Hofstede et al., 2010). The COR theory further indicates that cultural contexts profoundly influence individuals’ perceptions of resource value, with collectivist cultures emphasizing organizational identification and belonging (Halbesleben et al., 2009). When organizational morale is low, teaching-research officers may experience psychological distress due to a lack of identification. To uphold collective interests and strengthen their sense of belonging, they may sacrifice personal interests, working harder to gain a sense of belonging and establish their value within the group, which maybe associated with enhanced occupational well-being.

This study presents organizational climate as a moderating variable to examine boundary conditions under which self-efficacy and work engagement are associated with occupational well-being. Findings indicate that organizational climate predominantly functions as a facilitator or inhibitor to self-efficacy, an individual characteristic. Moreover, compensating for deficiencies in organizational climate by increasing work engagement constitutes a passive compensatory mechanism with inherent risks. Therefore, promoting the occupational well-being of teaching-research officers in ethnic regions necessitates fostering confidence and heightened work engagement and systematically cultivating a favorable, supportive organizational climate. Future educational management practices should prioritize establishing a “resource caravan channel” rather than expecting unlimited individual sacrifice. This is consistent with Nerstad et al. (2019).

## 6 Limitations and implications

Four limitations of this study warrant attention and should guide future research:

This study focuses on teaching-research officers in China’s ethnic minority regions, and caution is warranted when generalizing its findings. First, the sample primarily originates from a province in Southwest China, where specific regional cultures, educational policies, and developmental levels may limit the applicability of conclusions to other regions of China (particularly the more developed eastern regions) or to educational systems in other countries. Additionally, teaching-research officers occupy a unique dual role within China’s education system, combining administrative and professional responsibilities. Their work context differs from that of regular teachers or university faculty. Therefore, this model may require re-evaluation when applied to other educational groups or cultural contexts. Second, this study employs a cross-sectional design, which effectively reveals the associative patterns and moderating mechanisms among variables but cannot establish strict causal relationships. For instance, self-efficacy and occupational well-being may exhibit bidirectional influences or be jointly driven by other unmeasured variables. Future research may adopt longitudinal tracking designs or experimental intervention methods to more clearly elucidate the causal pathways among these variables. Third, this study preliminarily examined the overall mediating role of work engagement on the relationship between self-efficacy and occupational well-being. Future research may further investigate the mediating effects of the specific dimensions of work engagement: vitality, dedication, and focus. Alternatively, future research could introduce additional mediating or moderating variables, such as psychological resilience and emotional intelligence, between self-efficacy and occupational well-being to identify factors that empower highly self-efficacious teaching-research officers to maintain well-being in the face of adversity. Fourth, future research methodologies may integrate qualitative approaches, including in-depth interviews and case studies, to explore the challenges and psychological experiences of high-self-efficacy teaching-research officers functioning in low organizational climate settings. This would yield richer, contextually grounded interpretations to complement quantitative findings.

Despite its limitations, this study’s theoretical value and practical significance are noteworthy. Previous studies have predominantly focused on the impact of teachers’ self-efficacy on their occupational well-being. Therefore, this is the inaugural investigation into the correlation between self-efficacy and occupational well-being among teaching-research officers in primary and secondary schools in China’s ethnic minority regions, addressing a deficiency in professional well-being research. Theoretically, investigating the mechanisms of self-efficacy on the professional well-being of teaching-research officers in China’s ethnic minority regions holds significant importance. It improves our comprehension of the antecedents affecting their occupational well-being. It underscores the significant influence of self-efficacy on occupational well-being, providing a theoretical foundation for implementing interventions. Thus, this study enhances theoretical understanding by validating the relevance of positive psychology in educational management in China and broadening the concept of self-efficacy to encompass teaching-research officers who possess administrative and professional roles (Shen, 2013). This affirms self-efficacy as a fundamental psychological resource with distinct value for improving occupational well-being in China’s educational management context. Simultaneously, our research reveals how self-efficacy, work engagement, and organizational climate affect the occupational well-being of educational teaching-research officers. This provides a novel perspective and theoretical support for understanding methods to improve occupational well-being while broadening the applicability of resource conservation theory. From a practical standpoint, this study offers significant implications for educational administrators in China’s ethnic minority regions seeking to enhance the occupational well-being of K-12 teaching-research officers. Findings indicate that the occupational well-being of teaching-research officers in ethnic minority areas depends on their individual self-efficacy levels and requires coordinated support from educational administrative departments, schools, and other organizational stakeholders. Developing and improving the organizational climate within respective institutions is essential to providing employees with higher job satisfaction (Anderson et al., 2000; Duggan, 2008; Woods and Weasmer, 2002). Strengthen resource support and safeguards for teaching-research officers. For instance, dedicated funds for instructional research could be established, considering current research-related requirements for teaching-research officers, who focus on securing government-funded research projects. Practice-oriented research projects should be created (Yin et al., 2020), encouraging their participation in research-related activities (Van der Klink et al., 2017) and mobilizing their enthusiasm. Simultaneously, granting teaching-research officers autonomy in conducting teacher training fosters a harmonious work environment and provides opportunities for professional learning, thereby enhancing their sense of purpose and well-being. These measures strengthen the development of teacher education teams in underdeveloped regions and effectively elevate the overall educational quality of the region and the nation.

## 7 Conclusion

Our study represents the first exploration of the influencing factors and underlying mechanisms of occupational well-being among teaching-research officers within China’s ethnic minority regions, as well as the boundary conditions under which self-efficacy influences occupational well-being, thereby deepening theoretical understanding. Findings reveal: First, teaching-research officers’ self-efficacy is significantly positively correlated with occupational well-being. This indicates that higher levels of self-efficacy correlate with higher levels of occupational well-being. Second, work engagement mediates the relationship between self-efficacy and occupational well-being among teaching-research officers. This suggests that self-efficacy enhances work engagement, thereby increasing occupational well-being. Third, organizational climate moderates the strength of the relationship between self-efficacy and occupational well-being. Specifically, compared to low organizational climate conditions, the positive impact of self-efficacy on occupational well-being is more pronounced under high organizational climate conditions. Fourth, organizational climate also moderates the strength of the relationship between work engagement and occupational well-being. Specifically, compared to high organizational climate conditions, the positive impact of work engagement on job satisfaction is more pronounced for teaching-research officers in low organizational climate settings. These findings extend beyond the immediate Chinese context to contribute to international scholarly dialogues. Theoretically, they validate and refine COR theory and social cognitive theory by demonstrating their applicability within a specific, resource-constrained cultural and professional ecosystem. They underscore that the interplay between personal agency (self-efficacy), active resource investment (work engagement), and environmental support (organizational climate) is a fundamental dynamic influencing educator well-being across diverse settings. The implications for practice are both local and global. For educational administrators in China’s ethnic regions, the results argue against relying solely on individual resilience or sacrifice. Instead, they call for integrated support strategies that concurrently cultivate personal efficacy through targeted professional development, protect and stimulate intrinsic work engagement, and proactively build supportive organizational climates characterized by trust, clarity, and shared purpose. This integrated approach is essential for promoting sustainable professional flourishing. For policymakers and educational leaders in other nations facing similar challenges of resource disparity, teacher support, or educational equity, this study offers a transferable framework. It emphasizes that investing in systemic environments that nurture both individual and collective resources is not merely ancillary but central to sustaining educator well-being and, by extension, achieving long-term educational quality. Ultimately, safeguarding the well-being of those tasked with teacher educators is a universal imperative for building robust and equitable educational systems worldwide.

## Data Availability

The original contributions presented in the study are included in the article/supplementary material, further inquiries can be directed to the corresponding author.
